# 3D-Subspace-Based Auto-Paired Azimuth Angle, Elevation Angle, and Range Estimation for 24G FMCW Radar with an L-Shaped Array

**DOI:** 10.3390/s18041113

**Published:** 2018-04-05

**Authors:** HyungSoo Nam, Ying-Chun Li, ByungGil Choi, Daegun Oh

**Affiliations:** 1 Collaborative Robots Research Center, Daegu Gyeongbuk Institute of Science and Technology, Daegu 42988, Korea; hsnam@dgist.ac.kr (H.N.); choibk@dgist.ac.kr (B.C.); 2 Department of Electronic Engineering, Hanyang University, Seoul 04763, Korea; davis.y.lee@hotmail.com

**Keywords:** L-shaped array, FMCW, 3D subspace

## Abstract

In this paper, a three-dimensional (3D)-subspace-based azimuth angle, elevation angle, and range estimation method with auto-pairing is proposed for frequency-modulated continuous waveform (FMCW) radar with an L-shaped array. The proposed method is designed to exploit the 3D shift-invariant structure of the stacked Hankel snapshot matrix for auto-paired azimuth angle, elevation angle, and range estimation. The effectiveness of the proposed method is verified through a variety of experiments conducted in a chamber. For the realization of the proposed method, K-band FMCW radar is implemented with an L-shaped antenna.

## 1. Introduction

Recently, many commercialized radar systems have become available for automotive, surveillance, anti-drone, medical, and personal security applications, as in [1,2]. As the need for inexpensive high-performance radar is increasing, frequency-modulated continuous waveform (FMCW) radar has become popular due to its inherent ability to utilize a large bandwidth with a low sampling rate [3,4,5]. Moreover, a homodyne receiver structure for the RF (radio frequency) frequency of FMCW radar can be easily implemented with a mixer. We call this transformation via a mixer de-chirping. A variety of estimation algorithms for FMCW radar have been developed, such as range estimation [6,7], range and Doppler estimation [8,9], and range and azimuth angle estimation [10,11]. Among the literature [6,7,8,9,10,11], there are no 3D-subspace-based algorithms for the joint estimation of range, azimuth angle, and elevation angle in FMCW radars. At the same time, there is no implementation for an FMCW radar system with an L-shaped receiving antenna array. In particular, concerning the joint estimation of azimuth and elevation angle, an L-shaped antenna structure has been proposed with two-dimensional (2D) estimation algorithms [12,13] for paired estimation of the two angles in recent studies. Although the effectiveness of the suggested algorithms [10,11,12,13] has been demonstrated through simulations, they have not been verified by the implemented system and corresponding experiments. However, the proposed 3D-subspace-based algorithm for joint estimation of range, elevation angle, and azimuth angle is verified through experiments using the implemented FMCW radar system with an L-shaped receiving array. A variety of experiments have been done to demonstrate the effectiveness of the proposed algorithm with L-shaped receiving antennas.

In this paper, a 3D-subspace-based joint azimuth, elevation, and range estimation algorithm is proposed with a 3D shift-invariant stacked Hankel matrix, which consists of one-dimensional (1D) Hankel matrices in a specific way to make use of the phase relationship between the receiving channels in horizontally placed antennas and vertically placed antennas, respectively. In addition to the 3D shift-invariant parameter estimation algorithm, a 24 GHz FMCW radar system has been implemented with transmitting lens antenna and receiving L-shaped antenna elements. The effectiveness of the proposed algorithm was verified through a variety of experiments with the implemented FMCW radar system.

## 2. System Model

**Figure 1:**
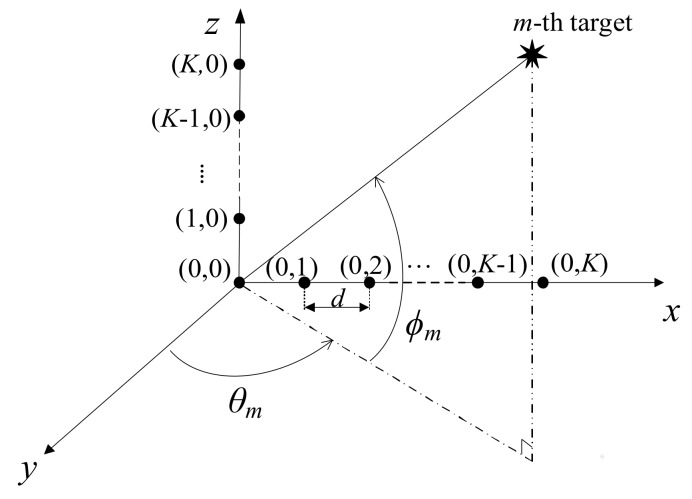
Basic L-shaped radar system scenario.

The transmitted FMCW chirp signal can be modeled from [3] by:

(1) $$s\left(t\right)\;=\;\left\{ \begin{array}{l} \exp\;\left(j\;\left(\omega_{c}t\;+\;\frac{\mu }{2}t^{2}\right)\right)\\ 0 \end{array}\right.\;\begin{array}{c} \mathrm{for}\;0\;\leq \;t\;<\;T_{sym}\;\\ \mathrm{elsewhere} \end{array}$$

where *ω_c_* denotes the carrier frequency, *μ* is the rate of change of the instantaneous frequency of a chirp signal, and *T_sym_* is the duration of chirp signals. Then, the bandwidth of the FMCW chirp signal is defined by *B* = *μT_sym_*/2π. Consider *M* far-field, non-coherent, narrowband sources impinging on the L-shaped array with 2*K* + 1 omnidirectional sensors as shown in Figure 1. The array on the *x-* and *z*-axes consists of two uniform linear sub-arrays with element spacing *d*, each being composed of *K* + 1 elements.

Let *ϕ_m_*, *θ_m_*, and *τ_m_* denote the elevation angle, azimuth angle, and the time delay of the *m*-th target, respectively. Then, the received FMCW signals at each antenna element can be represented by

(2) $$r_{p,q}(t)=\sum_{m=0}^{M-1}a_{m}\exp\left(jp\alpha_{m}\right)\exp\left(jq\beta_{m}\right)s\left(t-\tau_{m}\right)+w_{p,q}(t)$$

where *a_m_* denote the complex amplitude for the *m*-th target, (*p*,*q*)∈{(*K*,0), …, (1,0), (0,0), (0,1), …, (0,*K*)}, *M* denotes the number of targets, and *w_p,q_*(*t*) is the additive white Gaussian noise (AWGN) signal. As in Equation (2), it is assumed that the received signals are perturbed by only an AWGN source. Thus, there is no consideration for the non-Gaussian noise sources as in [14,15]. However, in practice, non-Gaussian noises occur frequently in realistic outdoor environments. Since the proposed algorithm is developed from the assumption of AWGN noise, it is impossible for the proposed method to be applied with the non-Gaussian model directly. However, it is possible to make use of a pre-whitening technique as a preprocessing step for the proposed algorithm in the case of the non-Gaussian noise model as in [16].

The two electrical angles α*_m_* and *β_m_* in Equation (2) can be represented from [9] by

(3) $$\begin{array}{l} \alpha_{m}=-\frac{2\pi }{\lambda_{s}}d\;\sin\;\phi_{m}\cos\;\theta_{m}\;\\ \beta_{m}=-\frac{2\pi }{\lambda_{s}}d\;\cos\;\phi_{m} \end{array}$$

where *λ_s_* denotes the wavelength of the carrier signal, *d*: element spacing.

In FMCW radar, the received FMCW chirp signals can be easily transformed into a sinusoidal waveform via a mixer and a low-pass filter in the RF circuit [1]. We call these sinusoids beat signals. The beat signals, transformed from the received FMCW signals of (2), can be represented as in [1] by

(4) $$y_{p,q}\left(t\right)=\left(\sum_{m=0}^{M-1}a_{m}\exp\left(jp\alpha_{m}\right)\exp\left(jq\beta_{m}\right)\exp\left(j\left(\mu \tau_{m}t-\frac{\mu }{2}\tau_{m}{}^{2}+\omega_{c}\tau_{m}\right)\right)\right)+{\bar{w}}_{p,q}(t)+z\left(t\right)$$

where ${\bar{w}}_{p,q}(t)$ denotes the transformed AWGN signal, and *z*(*t*) denotes the local oscillator (LO) phase noise of the mixer, which is independent from receiving channel indexes (*p*,*q*). After analog-to-digital conversion, the discrete time model of (4) with sampling frequency *f_s_* = 1/*T_s_* (*T_s_*: the sampling duration) satisfying the Nyquist criterion can be derived by *y_p,q_*[*n*] = *y_p,q_*(*nT_s_*) for *n* = 0, ..., *N* − 1 where *N* = *T_sym_*/*T_s_*.

**Figure 2:**
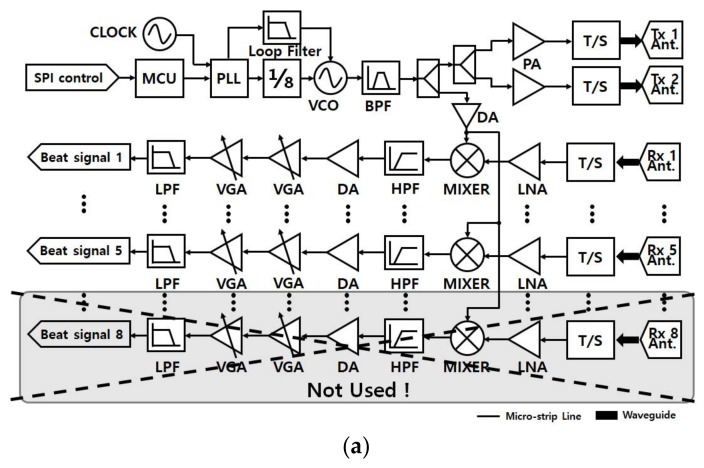

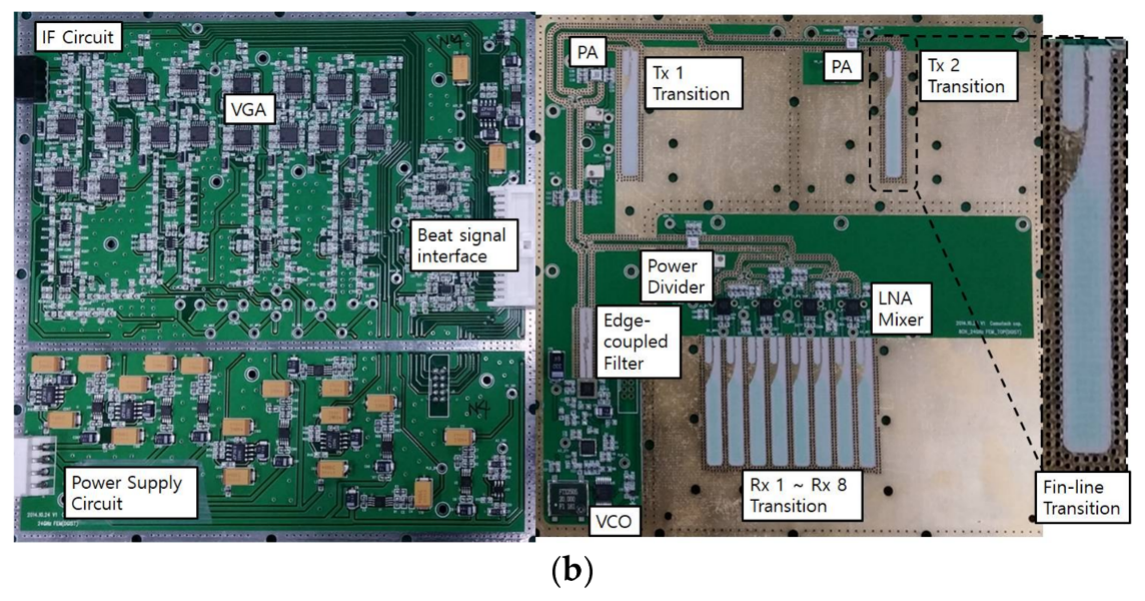
(**a**) Block diagram of the 24-GHz transceiver and IF; (**b**) Photograph of the 24-GHz transceiver and IF. PLL = phase-locked loop; VCO = voltage-controlled oscillator; LNA = low-noise amplifier, MCU = micro controller unit, SPI = serial peripheral interface, DA = driver amplifier, PA = driver amplifier, VGA = variable gain amplifier, HPF = high pass filter, BPF = band pass filter, LPF = low pass filter.

From [17], it was revealed that the LO phase noise is transferred to the output of the mixer. As depicted in Figure 2a, the LO signals of the implemented FMCW RF module are divided and transferred to the mixers of the five receiving channels. Thus, it can be said that the receiving five channels have the same phase noise *z*(*t*). Since the proposed method is designed to make use of phase difference between receiving channels for the estimation of elevation and azimuth angle, the LO phase noise cannot make any kind of disturbance on the estimation of azimuth and elevation angle due to the identical *z*(*t*) for all of the receiving channels. However, in respect of range estimation, *z*(*t*) can lead to some negative effects. In the implemented RF system, Temperature Compensate X’tal (crystal) Oscillator (TCXO), AST3TQ-50, which is used to generate the LO signal, shows the phase noise performance of −95 dBc/Hz at 10 Hz offset. Considering this phase noise characteristic, the perturbation of LO on range estimation is expected to be very small.

## 3. Proposed Algorithm

Since the proposed method has been developed for the joint estimation of elevation angle, azimuth angle, and range for FMCW radar with an L-shaped array, we propose a stacked Hankel matrix to exploit the 3D shift-invariant structure. Prior to explaining the 3D shift-invariant structure, the single shift-invariant structure in the temporal domain for range estimation is addressed with a mathematical factorization model. Then, the shift-invariant structure in the temporal domain is extended to the 3D shift-invariant structure for joint estimation of elevation, azimuth, and range in the spatial and temporal domains.

### 3.1. Shift-Invariant Structure for Range

For convenience in expressing the single shift-invariant structure for elevation, azimuth, and range, we assume that the received signals are not perturbed by AWGN. Noise perturbation is addressed later in conjunction with singular value decomposition (SVD).

Using the beat signals of the *p*-th and *q*-th antennas, *y_p,q_*[*n*] for *n* = 0, …, *N* − 1, the Hankel snapshot matrix can be defined as

(5) $$Y_{p,q}=\left[\begin{array}{cccc} y_{p,q}[0] & y_{p,q}[1] & \cdots & y_{p,q}[L_{r}-1]\\ y_{p,q}[1] & y_{p,q}[2] & \cdots & y_{p,q}[L_{r}]\\ \vdots & \vdots & \ddots & \vdots \\ y_{p,q}[L_{c}-1] & y_{p,q}[L_{c}] & \cdots & y_{p,q}[N-1] \end{array}\right]$$

where *L_r_* and *L_c_* = *N* − *L_r_* + 1 are the selection parameters, which satisfy the conditions *L_r_* ≥ *M* and *L_c_* ≥ *M*. Without considering the perturbation by AWGN, the Hankel snapshot matrix ***Y****_p_*_,*q*_ can be factorized as in [18] by

(6) $$Y_{p,q}=AHR_{p,q}B$$

(7) $$\begin{array}{c} \mathrm{Where}\;A=\left[\begin{array}{cccc} 1 & 1 & \cdots & 1\\ \kappa_{0} & \kappa_{1} & \cdots & \kappa_{M-1}\\ \vdots & \vdots & \ddots & \vdots \\ \kappa_{0}^{L_{c}-1} & \kappa_{1}^{L_{c}-1} & \cdots & \kappa_{M-1}^{L_{c}-1} \end{array}\right],B=\left[\begin{array}{cccc} 1 & \kappa_{0} & \cdots & \kappa_{0}^{L_{r}-1}\\ 1 & \kappa_{1} & \cdots & \kappa_{1}^{L_{r}-1}\\ \vdots & \vdots & \ddots & \vdots \\ 1 & \kappa_{M-1} & \cdots & \kappa_{M-1}^{L_{r}-1} \end{array}\right],\\ H=\left[\begin{array}{ccc} a_{0}\exp\left(j\left(-\frac{\mu }{2}\tau_{0}{}^{2}+\omega_{c}\tau_{0}\right)\right) & \cdots & 0\\ \vdots & \ddots & \vdots \\ 0 & \cdots & a_{M-1}\exp\left(j\left(-\frac{\mu }{2}\tau_{M-1}{}^{2}+\omega_{c}\tau_{M-1}\right)\right) \end{array}\right],\\ \mathrm{and}\;R_{p,q}=\left[\begin{array}{ccc} \exp\left(jp\alpha_{0}\right)\exp\left(jq\beta_{0}\right) & \cdots & 0\\ \vdots & \ddots & \vdots \\ 0 & \cdots & \exp\left(jp\alpha_{M-1}\right)\exp\left(jq\beta_{M-1}\right) \end{array}\right] \end{array}$$

In (7), *κ_m_* is the delay-induced phase shift between the adjacent samples, such that

(8) $$\kappa_{m}=\exp\;\left(j\mu \tau_{m}T_{s}\right)$$

In (6), the diagonal matrices ***H*** and ***R****_p_*_,*q*_ are composed of the phase terms of the beat signals of (4). The Vandermonde structured matrices ***A*** and ***B*** are defined in terms of the phase shifts *κ*_0_, …, *κ_M_*_− 1_, which are not changed by array indices *p* and *q*.

From ***Y****_p_*_,*q*_ of (5), the two sub-matrices ***Y****_p_*_,*q*,0_ and ***Y****_p_*_,*q*,1_ can be defined by

(9) $$Y_{p,q,0}=\left[\begin{array}{cccc} y_{p,q}[0] & y_{p,q}[1] & \cdots & y_{p,q}[L_{r}-1]\\ y_{p,q}[1] & y_{p,q}[2] & \cdots & y_{p,q}[L_{r}]\\ \vdots & \vdots & \ddots & \vdots \\ y_{p,q}[L_{c}-2] & y_{p,q}[L_{c}] & \cdots & y_{p,q}[N-2] \end{array}\right]$$

(10) $$Y_{p,q,1}=\left[\begin{array}{cccc} y_{p,q}[1] & y_{p,q}[2] & \cdots & y_{p,q}[L_{r}]\\ y_{p,q}[2] & y_{p,q}[3] & \cdots & y_{p,q}[L_{r}+1]\\ \vdots & \vdots & \ddots & \vdots \\ y_{p,q}[L_{c}-1] & y_{p,q}[L_{c}] & \cdots & y_{p,q}[N-1] \end{array}\right]$$

Comparing ***Y****_p_*_,*q*,0_ with ***Y****_p_*_,*q*,1_, it can be easily seen that ***Y****_p_*_,*q*,1_ is the shifted version of ***Y****_p_*_,*q*,0_ in the column direction. Based on the factorization model of (6), the sub-matrices ***Y****_p_*_,*q*,0_ and ***Y****_p_*_,*q*,1_ can also be factorized, such that

(11) $$\begin{array}{l} Y_{p,q,0}=A_{0}HR_{p,q}B\\ Y_{p,q,1}=A_{0}\Sigma HR_{p,q}B \end{array}$$

(12) $$\mathrm{Where}\;A_{0}=\left[\begin{array}{cccc} 1 & 1 & \cdots & 1\\ \kappa_{0} & \kappa_{1} & \cdots & \kappa_{M-1}\\ \vdots & \vdots & \ddots & \vdots \\ \kappa_{0}^{L_{c}-2} & \kappa_{1}^{L_{c}-2} & \cdots & \kappa_{M-1}^{L_{c}-2} \end{array}\right]\;\mathrm{and}\;\Sigma =\left[\begin{array}{cccc} \kappa_{0} & 0 & \cdots & 0\\ 0 & \kappa_{1} & \cdots & 0\\ \vdots & \vdots & \ddots & \vdots \\ 0 & 0 & \cdots & \kappa_{M-1} \end{array}\right]$$

In (11), it is noteworthy that factorizations for ***Y****_p_*_,*q*,0_ and ***Y****_p_*_,*q*,1_ are the same except for the diagonal matrix ***Σ*** of ***Y****_p_*_,*q*,1_. We call this a shift-invariant structure. This single shift-invariant relationship between ***Y****_p_*_,*q*,0_ and ***Y****_p_*_,*q*,1_ in (11) is extended to a 3D shift-invariant structure for the joint estimation of range with elevation angle and azimuth angle.

### 3.2. Shift-Invariant Structure for Two Electrical Angles

The stacked Hankel snapshot matrix for the joint estimation of range and azimuth and elevation angles can be defined by

(13) $$Y=\left[\begin{array}{l} Y_{0,0}\\ Y_{X}\\ Y_{Y} \end{array}\right],\;\mathrm{where}\;Y_{X}=\left[\begin{array}{c} Y_{1,0}\\ Y_{2,0}\\ \vdots \\ Y_{K,0} \end{array}\right]\;\mathrm{and}\;Y_{Y}=\left[\begin{array}{c} Y_{0,1}\\ Y_{0,2}\\ \vdots \\ Y_{0,K} \end{array}\right]$$

In (13), the matrix ***Y***_0,0_ takes the role of reference for pairing afterwards. Prior to explaining the 3D shift-invariant structure in ***Y***, the shift-invariant structure for two electronic angles α*_m_* and *β_m_* is addressed.

Concerning α*_m_*, the two stacked matrices, which are sub-matrices of ***Y_X_***, can be defined as

(14) $$Y_{X\_0}=\left[\begin{array}{c} Y_{1,0}\\ Y_{2,0}\\ \vdots \\ Y_{K-1,0} \end{array}\right]\;\mathrm{and}\;Y_{X\_1}=\left[\begin{array}{c} Y_{2,0}\\ Y_{3,0}\\ \vdots \\ Y_{K,0} \end{array}\right]$$

Then, based on the factorization of (6), ***Y****_X_*__0_ and ***Y****_X_*__1_ can be rewritten as

(15) $$Y_{X\_0}=\left[\begin{array}{c} AHR_{1,0}B\\ AHR_{2,0}B\\ \vdots \\ AHR_{K-1,0}B \end{array}\right]\;\mathrm{and}\;Y_{X\_1}=\left[\begin{array}{c} AHR_{2,0}B\\ AHR_{3,0}B\\ \vdots \\ AHR_{K,0}B \end{array}\right]$$

Since the diagonal matrix ***R****_p_*_,*q*_ is composed of the two electronics angles α*_m_* and *β_m_*, it is not convenient to carry out further factorization in (15). Thus, we define the two diagonal matrices ***Θ*** and ***Φ***, whose elements are only composed of α*_m_* or *β_m_*, respectively, such that

(16) $$\Theta =\left[\begin{array}{ccc} \exp\left(j\alpha_{0}\right) & \cdots & 0\\ \vdots & \ddots & \vdots \\ 0 & \cdots & \exp\left(j\alpha_{M-1}\right) \end{array}\right]$$

(17) $$\Phi =\left[\begin{array}{ccc} \exp\left(j\beta_{0}\right) & \cdots & 0\\ \vdots & \ddots & \vdots \\ 0 & \cdots & \exp\left(j\beta_{M-1}\right) \end{array}\right]$$

Then, we can derive the following relationship: ***R****_k_*_,0_ *= **R**_k_*_−1,0_***Θ***. Using this, ***Y****_X_*__0_ and ***Y****_X_*__1_ of (15) can be reformulated as

(18) $$\begin{array}{l} Y_{X\_0}=\left[\begin{array}{c} AHR_{1,0}B\\ AHR_{1,0}\Theta B\\ \vdots \\ AHR_{1,0}\Theta^{K-2}B \end{array}\right]\\ Y_{X\_1}=\left[\begin{array}{c} AHR_{1,0}\Theta B\\ AHR_{1,0}\Theta^{2}B\\ \vdots \\ AHR_{1,0}\Theta^{K-1}B \end{array}\right] \end{array}$$

Applying the generalized eigenvalue decomposition (EVD) relationship for ***Y****_X_*__0_ and ***Y****_X_*__1_ such that (***Y****_X_*__1_ − *λ****Y****_X_*__0_)***β*** = ***0,*** where *λ* and ***β*** denote the eigenvalue and the corresponding eigenvector, respectively, this EVD can be redefined using (18) by

(19) $$\left(\left[\begin{array}{c} AHR_{0,0}\Theta B\\ AHR_{0,0}\Theta^{2}B\\ \vdots \\ AHR_{0,0}\Theta^{K}B \end{array}\right]-\lambda \left[\begin{array}{c} AHR_{0,0}B\\ AHR_{0,0}\Theta B\\ \vdots \\ AHR_{0,0}\Theta^{K-1}B \end{array}\right]\right)\beta =0\Leftrightarrow \left(\left[\begin{array}{c} AHR_{0,0}\left[\Theta -\lambda I_{M}\right]Q\\ AHR_{0,0}\Theta \left[\Theta -\lambda I_{M}\right]Q\\ \vdots \\ AHR_{0,0}\Theta^{K-1}\left[\Theta -\lambda I_{M}\right]Q \end{array}\right]\right)\beta =0$$

In (19), it is straightforward that the rank of (***Y****_X_*__1_ − *λ****Y****_X_*__0_) will be *M* unless *λ* = α*_m_* for *m* = 0,1, …, *M* − 1, since *M* sources are assumed in (2). However, when *λ* = α*_m_* for *m* = 0, …, *M* − 1, the rank of (***Y****_X_*__1_ − *λ****Y****_X_*__0_) will be *M* − 1 since one of the rows in [(***Θ*** − *λ****I****_M_*)] will be zero. This means that we can obtain α*_m_* as a result of the generalized EVD of ***Y****_X_*__1_ and ***Y****_X_*__0_.

Similar to the derivation of the EVD relationship for α*_m_* in Equations (14)–(19), the EVD relationship for *β_m_* can be derived as follows. Two matrices, which are also sub-matrices of ***Y_Y_***, can be defined as

(20) $$\begin{array}{l} Y_{Y\_0}=\left[\begin{array}{c} Y_{0,1}\\ Y_{0,2}\\ \vdots \\ Y_{0,K-1} \end{array}\right]\\ Y_{Y\_1}=\left[\begin{array}{c} Y_{0,2}\\ Y_{0,3}\\ \vdots \\ Y_{0,K} \end{array}\right] \end{array}$$

Concerning *β_m_*, it satisfies ***R***_0,*k*_ = ***R***_0,*k*−1_***Φ***. Then, ***Y****_Y_*__0_ and ***Y****_Y_*__1_ of (20) can be factorized and reformulated by

(21) $$\begin{array}{l} Y_{Y\_0}=\left[\begin{array}{c} AHR_{0,1}B\\ AHR_{0,0}\Phi B\\ \vdots \\ AHR_{0,1}\Theta^{K-2}B \end{array}\right]\\ Y_{Y\_1}=\left[\begin{array}{c} AHR_{0,1}\Phi B\\ AHR_{0,0}\Phi^{2}B\\ \vdots \\ AHR_{0,0}\Phi^{K-1}B \end{array}\right] \end{array}$$

Applying the generalized EVD relationship for ***Y****_Y_*__0_ and ***Y****_Y_*__1_ such that (***Y****_Y_*__1_ − *λ****Y****_Y_*__0_)***β*** = ***0***, it is also straightforward that the rank of (***Y****_Y_*__1_ − *λ****Y****_Y_*__0_) will be *M* unless *λ* = *β_m_* for *m* = 0,1, …, *M* − 1. When *λ* = *β_m_* for *m* = 0, …, *M* − 1, the rank of (***Y****_Y_*__1_ − *λ****Y****_Y_*__0_) will be *M* − 1 since one of the rows in ((***Φ*** − *λ****I****_M_*)) will be zero.

Concerning the generalized EVD form about *κ_m_* for the given stacked matrix ***Y***, two sub-matrices ***Y***_0_ and ***Y***_1_ can be defined to make use of the shift-invariant structure in (11) and (12) by

(22) $$\begin{array}{l} Y_{0}=J_{0}Y\\ Y_{1}=J_{1}Y\; \end{array}$$

where the selection matrices.

(23) $$\begin{array}{l} J_{0}=I_{2K+1}⊗\left[I_{L_{c}-1}\;0_{L_{c}-1\times 1}\right]\\ J_{1}=I_{2K+1}⊗\left[0_{L_{c}-1\times 1}\;I_{L_{c}-1}\right] \end{array}$$

where ***I****_k_* denotes the identity matrix of *k* by *k*, and ***0****_m_*_×_*_n_* denotes the zero matrix of *m* by *n*. Then, the generalized EVD relationship for ***Y***_0_ and ***Y***_1_ such that (***Y***_1_ − *λ****Y***_0_)***β*** = ***0*** can lead to *λ* = *κ_m_* for *m* = 0,1, …, *M* − 1 based on (11).

### 3.3. Signal Subspace

In the preceding section, a noiseless environment in ***Y*** was assumed for the derivation of the EVD relationships in association with *κ*_m_, α*_m_,* and *β_m_*. Considering perturbation of AWGN on the received signals, first, singular value decomposition (SVD) should be applied with ***Y*** to separate the signal subspace from the noise subspace. In the presence of AWGN, the stacked matrix ***Y*** can be factorized by SVD into signal and noise subspaces as in [19], such that

(24) $$Y=U\Sigma V^{T}=\left[\begin{array}{cc} U_{s} & U_{n} \end{array}\right]\left[\begin{array}{cc} \Sigma_{s} & \\ & \Sigma_{n} \end{array}\right]{\left[\begin{array}{cc} V_{s} & V_{s} \end{array}\right]}^{T}=\underset{\mathrm{signal}\;\mathrm{subspace}}{\underset{︸}{U_{s}\Sigma_{s}V_{s}^{T}}}+\underset{\mathrm{noise}\;\mathrm{subspace}}{\underset{︸}{U_{n}\Sigma_{n}V_{n}^{T}}}$$

where ***U*** and ***V*** are the orthogonal matrix, ***Σ*** denotes the diagonal matrix with singular values in the diagonal elements, ***U****_s_*, ***Σ****_s_*, and ***V****_s_* are associated with the signal subspace, and ***U****_n_*, ***Σ****_n_*, and ***V****_n_* are associated with the noise subspace. Although ***U****_s_* and ***U****_n_* are modeled to be separated with each other after SVD in (24), the signal subspace and the noise subspace cannot be separated with each other through only SVD. Generally, all of the subspace-based estimation algorithms [12,13,14,15,16,18,19] essentially involve the subspace classification step, in which the signal subspace and the noise subspace are separated from each other. In this step, SVD or EVD is used for matrix decomposition and its singular- or eigenvalues are used with the Akaike Information Criterion (AIC) or the Minimum Descriptive Length (MDL) [20] for subspace separation. At this time, a high signal-to-noise ratio (SNR) is required with the subspace-based algorithms, which use the signal subspace or the noise subspace for correct subspace classification as in [18]. Thus, in a low SNR situation, performance degradation can be incurred by incorrect subspace classification.

Let us define a steering matrix ***Y****_S_*, which shows the shift-invariant structure in ***Y***, such that

(25) $$Y_{s}=\;\left[\begin{array}{c} AHR_{0,0}\\ AHR_{1,0}\\ \vdots \\ AHR_{K,0}\\ AHR_{0,1}\\ \vdots \\ AHR_{0,K} \end{array}\right]$$

Then, it satisfies [21]

(26) $$Y_{s}=U_{s}T$$

where ***T*** denotes full rank *M* by *M* transformation matrix. From ***U****_s_*, the pairs of sub-matrices such that {***U****_s,_*_0_, ***U****_s,_*_1_}, {***U****_s,X_*__0_, ***U****_s,X_*__1_}, and {***U****_s,Y_*__0_, ***U****_s,Y_*__1_} can be defined such that

(27) $$\begin{array}{l} U_{s,0}=J_{0}U_{s}\\ U_{s,1}=J_{1}U_{s} \end{array}$$

(28) $$\begin{array}{l} U_{s,X\_0}=J_{X\_0}U_{s}\\ U_{s,X\_1}=J_{X\_1}U_{s} \end{array}$$

(29) $$\begin{array}{l} U_{s,Y\_0}=J_{Y\_0}U_{s}\\ U_{s,Y\_1}=J_{Y\_1}U_{s} \end{array}$$

In (28), ***J****_X_*__0_ and ***J****_X_*__1_ are selection matrices, which can extract the sub-matrices from ***U****_s_* corresponding to ***Y****_X_*__0_ and ***Y****_X_*__1_, respectively, such that

(30) $$\begin{array}{l} J_{X\_0}=\left[0_{K-1,1}\;I_{K-1}\;0_{K,K+1}\right]⊗I_{L_{c}}\\ J_{X\_1}=\left[0_{K-1,2}\;I_{K-1}\;0_{K-1,K}\right]⊗I_{L_{c}} \end{array}$$

In (29), ***J****_Y_*__0_ and ***J****_Y_*__1_ are selection matrices, which can extract the sub-matrices from ***U****_s_* corresponding to ***Y****_Y_*__0_ and ***Y****_Y_*__1_, respectively, such that

(31) $$\begin{array}{l} J_{Y\_0}=\left[0_{K-1,K+1}\;I_{K-1}\;0_{K-1,1}\right]⊗I_{L_{c}}\\ J_{Y\_1}=\left[0_{K-1,K+2}\;I_{K-1}\right]⊗I_{L_{c}} \end{array}$$

In the preceding section, noiseless samples are assumed and used to derive the EVD relationships, (***Y***_1_ − *λ****Y***_0_)***β*** = ***0***, (***Y****_X_*__1_ − *λ****Y****_X_*__0_)***β*** = ***0***, and (***Y****_Y_*__1_ − *λ****Y****_Y_*__0_)***β*** = ***0***, for *κ_m_*, α*_m_*, and *β_m_*, respectively. However, noiseless samples cannot be made in a real RF system, so the sub-matrices from the signal subspace can substitute for the noiseless sub-matrices in three kinds of EVD forms for *κ_m_*, α*_m_*, and *β_m_*, as follows:

(32) $$\left(Y_{1}-\lambda Y_{0}\right)\beta =0\Rightarrow \left(U_{s,1}-\lambda U_{s,0}\right)\beta =0$$

(33) $$\left(Y_{X\_1}-\lambda Y_{X\_0}\right)\beta =0\Rightarrow \left(U_{s,X\_1}-\lambda U_{s,X\_0}\right)\beta =0$$

(34) $$\left(Y_{Y\_1}-\lambda Y_{Y\_0}\right)\beta =0\Rightarrow \left(U_{s,Y\_1}-\lambda U_{s,Y\_0}\right)\beta =0$$

### 3.4. Low-Complexity Pairing

The preceding section addressed how each of the three parameters α*_m_*, *β_m_*, and *κ_m_* can be estimated from ***U***_s_ in (32)–(34). However, how those estimates are paired to each other was not addressed, which must be handled for joint range, azimuth, and elevation estimation. In the literature [22,23,24,25,26,27], 2D parameter estimation algorithms have been proposed for angle and frequency in [22,23], range and Doppler in [24,25], and azimuth and elevation in [26,27]. All of the algorithms have their own mechanisms to pair the two kinds of estimated parameters for each target. In particular, to reduce the operation cost of paired estimation, computationally efficient algorithms have been proposed in [28,29]. However, all of the algorithms in [22,23,24,25,26,27,28,29] are limited to two-dimensional parameter estimation. In particular, when the number of parameters to be matched increases from two to three, the computational complexity greatly increases for 3D matching. In this paper, a low-complexity matching method using 1D EVD is proposed.

The generalized EVD forms in Equations (32)–(34) can be rewritten in the EVD form with a pseudo-inverse, such that

(35) $$G=U_{s,0}^{†}U_{s,1}=Β\Sigma {Β}^{-1}.$$

(36) $$G_{X}=U_{s,X\_0}^{†}U_{s,X\_1}={Β}_{X}\Theta {Β}_{X}^{-1},$$

(37) $$G_{Y}=U_{s,Y\_0}^{\;†}\;U_{s,Y\_1}={Β}_{Y}\;\Phi \;{Β}_{\;Y}^{\;-1},$$

where ***G***, ***G**_X_*, and ***G****_Y_* are *M*-by-*M* full-rank matrices, the superscript † denotes the corresponding Hermitian conjugate of a matrix, and ***B***, ***B****_X_*, and ***B****_Y_* involve the *M* eigenvectors corresponding to the eigenvalues in ***Σ***, ***Θ,*** and ***Φ***, respectively. Considering no perturbation of AWGN on signal subspace ***U***_s_, ***B***, ***B****_X_*, and ***B****_Y_* are identical to each other, since the pairs of the sub-matrices {***U****_s,_*_0_, ***U****_s,_*_1_}, {***U****_s,X_*__0_, ***U****_s,X_*__1_}, and {***U****_s,Y_*__0_, ***U****_s,Y_*__1_} are partitioned from only ***U***_s_, and the elements of the diagonal matrices ***Σ***, ***Θ,*** and ***Φ***, are modeled in ascending order.

Now, considering perturbation of AWGN on ***U***_s_, we first performed EVD on ***G****_X_* of Equation (35),

(38) $${\hat{G}}_{X}=U_{s,0}^{\;†}\;U_{s,1}=\hat{Β}\;\hat{\Theta }\;{\hat{Β}}^{\;-1}$$

Using the obtained $\hat{B}$, we performed diagonalization on ***G*** and ***G****_Y_*:

(39) $$\hat{\Sigma }\;=\;{\hat{Β}}^{-1}G\hat{Β}$$

(40) $$\hat{\Phi }\;=\;{\hat{Β}}^{\;-1}\;G_{Y}\;\hat{Β}$$

Through the derivation of (38)–(40), the values of estimated ${\hat{\kappa }}_{m}$, ${\hat{\alpha }}_{m}$, and ${\hat{\beta }}_{m}$ for the *m*-th target can be found in the diagonal line of matrices $\hat{\Sigma }$, $\hat{\Theta }$, and $\hat{\Phi }$ in ascending order, respectively. That is, for each *m*, the estimated values {${\hat{\kappa }}_{m}$, ${\hat{\alpha }}_{m}$, ${\hat{\beta }}_{m}$} are matched well for the *m*-th target by low complexity. Based on (3) and (8), the estimated elevation angle, azimuth angle, and the time delay of the *m*-th target can be expressed as

(41) $${\hat{\phi }}_{m}=-\mathrm{acos}\frac{\mathrm{angle}\left({\hat{\beta }}_{m}\right)\lambda_{s}}{2\pi d}$$

(42) $${\hat{\theta }}_{m}=-\mathrm{asin}\frac{\mathrm{angle}\left({\hat{\alpha }}_{m}\right)\lambda_{s}}{2\pi \mathrm{dcos}\left({\hat{\phi }}_{m}\right)}$$

(43) $${\hat{\tau }}_{m}=\frac{\mathrm{angle}\left({\hat{\kappa }}_{m}\right)}{2\pi \mu T_{s}}$$

where asin ($\frac{\mathrm{angle}\left({\hat{\alpha }}_{m}\right)\lambda_{s}}{2\pi d\cos\left({\hat{\phi }}_{m}\right)}$) denotes the inverse sine function, acos ($\frac{\mathrm{angle}\left({\hat{\beta }}_{m}\right)\lambda_{s}}{2\pi d}$) denotes the inverse cosine function, and angle (${\hat{\beta }}_{m}$), angle (${\hat{\alpha }}_{m}$), angle (${\hat{\kappa }}_{m}$)denotes the angle in radian, respectively.

### 3.5. Complexity Analysis

Since the proposed method is designed to estimate 3D parameters, such as range, azimuth, and elevation, it is fair to compare the computational complexity of the proposed method with that of the conventional 3D parameter estimation methods of an FMCW radar system. However, it was difficult to find relevant research results about 3D parameter estimation algorithms for an FMCW radar system, while 2D parameter estimation algorithms have been suggested for FMCW radar as in [11,30]. Thus, in this article, the computational complexity of the proposed algorithm is analyzed and compared with that of 2D multiple signal classification (MUSIC) of an FMCW radar system in [11,30]. The costs for the required operations are summarized in Table 1.

For the given data matrix ***Y*** of (13), the computational complexity costs for 2D MUSIC of [11,30] can be derived to be *O*(*K*^2^*L_c_* + *K*$L_{c}^{2}\;$+ $L_{c}^{3}$ + *b*^2^*K*^2^$L_{c}^{2}$), where *b* is the iteration number for two-dimensional searching. In general, the iteration number *b* is set to be much bigger than *K*, *L_c_*, and *M* for a high-resolution 2D pseudo-spectrum. Thus, using *b* >> *K*, *L_c_*, *M*, the derived complexity cost for 2D MUSIC can be simplified to *O*(*b*^2^*K*^2^$L_{c}^{2}$). For the proposed method, the computational complexity can be derived to *O*(*K*^2^*L_c_* + *K*$L_{c}^{2}\;$+ *L_c_*^3^ + *M*^3^ + 2*M*^2^*(L_c_* – 1*)* + 2*M*^2^(*K* – 1)), which can be approximated using *L_c_* >> *K* to *O*($L_{c}^{3}$ + *M*^3^ + *M*^2^*L_c_*)*.* Comparing *O*($L_{c}^{3}$ + *M*^3^ + *M*^2^*L_c_*) of the proposed method with *O*(*b*^2^*K*^2^$L_{c}^{2}$) of 2D MUSIC, we conclude that the cost of the proposed algorithm for 3D parameter estimation is much less than that of 2D MUSIC.

## 4. Implementation of 24-GHz L-Shaped Radar

To verify the effectiveness of the proposed method with L-shaped receiving antennas, a 24-GHz FMCW radar system with L-shaped receiving antennas was implemented. The implemented system consists of an antenna, an RF circuit, an IF circuit, a data-logging platform, and data-logging software. The data-logging platform takes the role of gathering received IF samples and passing them to the data-logging software in a personal computer (PC). The logged data is saved as files. Then, the proposed method, realized in MATLAB (The MathWorks, Natick, MA, USA), is applied with the saved data in the PC.

### 4.1. Transmitting and L-Shaped Receiving Antennas

**Figure 3:**
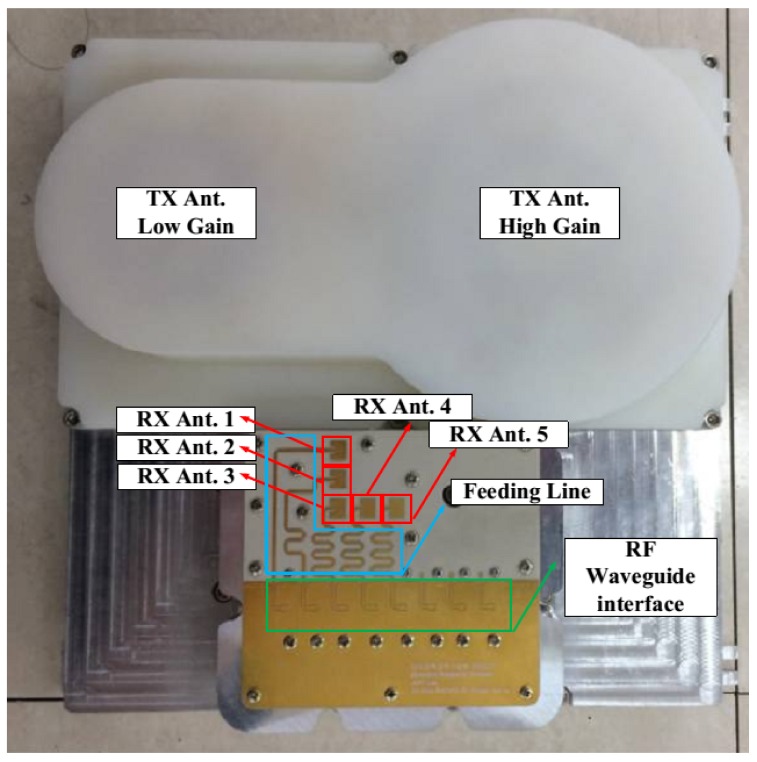
Transmitting lens antenna and receiving L-shaped antenna. TX: transmission, RF: radio frequency.

The transmitting antenna is composed of two lens antennas, which have 14-dBi and 19-dBi antenna gain. The 19-dBi high-gain antenna is selected as the default, and it is possible to change to the low-gain antenna by using the data-logging platform. The L-shaped receiving antenna is composed of five micro-strip patch antennas, designed to have about 6-dBi antenna gain, fabricated on an Ro4003 substrate with 0.012 inch thickness. The five receiving antennas have feeding lines with the same electric length and a meander structure as shown in Figure 3.

Figure 3 shows the configuration of the transmitting and receiving antennas; the transmitting antenna is located on the upper side of system, and the receiving antenna is on the lower side. The RF front module is designed to be able to obtain the signals from the receiving antennas through a rectangular waveguide interface.

The receiving patch antennas are set in an L-shaped formation for simultaneous elevation and azimuth angle estimation. The two electrical angles α*_m_* and *β_m_* of Equation (3) can be estimated using receiving antennas 1 to 3 and receiving antennas 3 to 5, respectively, as shown in Figure 1. Each patch is located by 1 wave length distance vertically or horizontally, resulting in a field of view of ±30° in azimuth and elevation.

**Figure 4:**
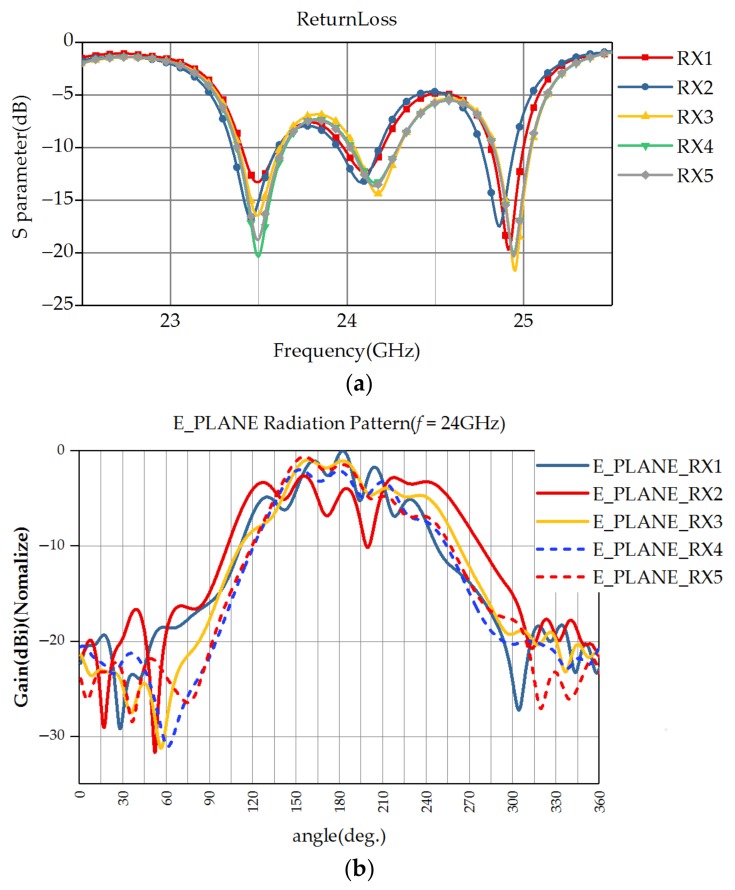
(**a**) Receiving antenna return loss; (**b**) Simulated radiation pattern (normalized).

The antenna operates over the frequency range of 24.025 to 24.225 GHz for the FMCW radar signal bandwidth as shown in Figure 4a. Figure 4b shows the simulated receiving antenna radiation pattern; in this case, the antenna is designed to face forward. The return loss and radiation pattern in Figure 4 are simulated together with the waveguide, transition, and meander feeding line. The antenna characteristic is optimized for radar performance with adjacent antennas and discontinuity of structure, although it is possible to improve the antenna characteristic.

**Figure 5:**
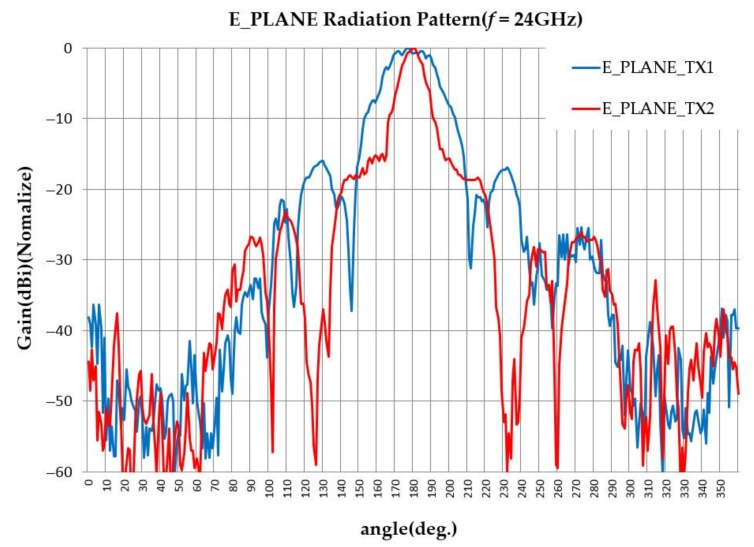
Measured transmission radiation pattern (normalized).

The transmitting antennas are designed by the lens to have a 12° beam width for the high-gain antenna and a 36° beam width for the low-gain antenna, and the side-lobe levels of both antennas are lower than 15 dB as shown in Figure 5.

The pattern was measured from the effective isotropic radiated power and normalized by the calculated transmit power by the RF system output power. The measured equivalent isotropically radiated power (EIRP) values were 39.3 dBm for the high-gain antenna and 34.1 dBm for the low-gain antenna.

### 4.2. 24-GHz Transceiver and IF

The 24-GHz FMCW RF system was implemented with a frequency synthesizer, a phase-locked loop (PLL) circuit, a voltage-controlled oscillator (VCO), an edge-coupled filter for band-pass filtering, a power amplifier (PA), and a low-noise amplifier (LNA). The overall structures of the RF and IF systems are described in Figure 2a.

Since we assume a saw-tooth wave as in (1), a PLL chip was set to generate a saw-tooth wave pattern with a 100-us period and a 200-MHz bandwidth over the range of 24.025 to 24.225 GHz with 10 dBm VCO output power. The reference clock of PLL is 20 MHz, which comes from TCXO. The VCO output is filtered with an edge-coupled filter to improve the side-lobe characteristic. As shown in Figure 2b, the edge-coupled filter is implemented with a center frequency of 24.125 GHz, a 10% bandwidth, and a Chebyshev-type band pass filter. The parameters of the implemented RF system are summarized in Table 2.

The filtered VCO signal is divided for transmission (TX) and LO as shown in Figure 2b. To increase the power of the divided VCO signal, a PA with a 20-dBm output P1dB is used. Since a WR34 interface is used with transmitting and receiving antennas, a fin-line structure transition is implemented to transform between the micro-strip line and WR34. The front side of the fin-line transition is connected to WR34, i.e., the signals on the micro-strip line are rotated 90° as shown in Figure 2b.

### 4.3. Data-Logging Platform

**Figure 6:**
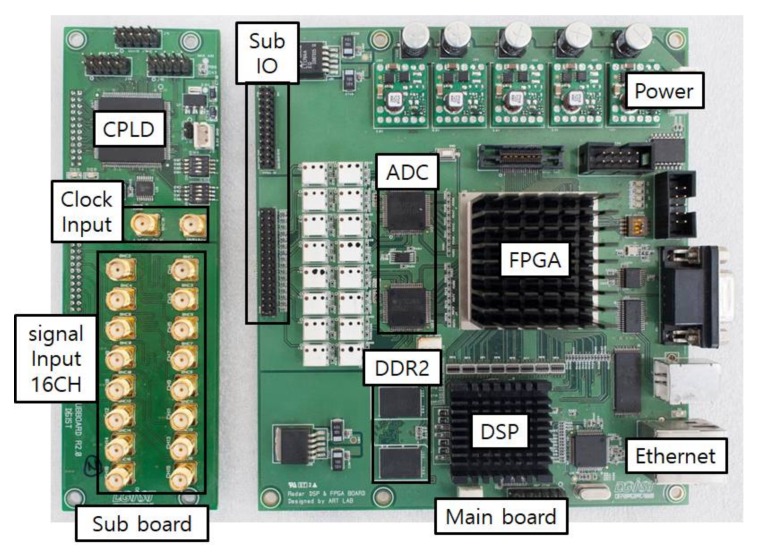
Photograph of the developed data-logging platform. IO: input output, CPLD: complex programmable logic device, ADC: analog to digital converter, DSP: digital signal processor, DDR2: double data rate two, FPGA: field programmable gate array.

The radar-logging platform was implemented to transfer maximum 16 channel analog digital converter (ADC) input signals to the PC in real-time. This platform mainly consists of digital signal processor (DSP) and field programmable gate array (FPGA) chips, namely TI TMS320C6455 and Stratix EP4SE530. The ADC output signals of a maximum of 16 channels are first saved in the first in first out (FIFO) memory of the FPGA and then transferred to the double-data-rate two synchronous dynamic random access memory (DDR2 SDRAM) through a direct memory access mechanism. The saved data can be handled by the DSP chip or transferred to the PC through 1 G local area network (LAN) communication as shown in Figure 6. In this work, the proposed algorithm is implemented in MATLAB and applied with the data saved in the PC by the data-logging platform. The specifications of the developed data-logging platform are summarized in Table 3.

## 5. Experiments

This section describes several indoor experiments, which were conducted to detect one target, two targets, and four targets, respectively, in the microwave anechoic chamber of Daegu Gyeongbuk Institute of Science & Technology (Daegu, Korea). One outdoor experiment was also carried out to verify the performance of the proposed algorithm in a realistic scenario.

**Figure 7:**
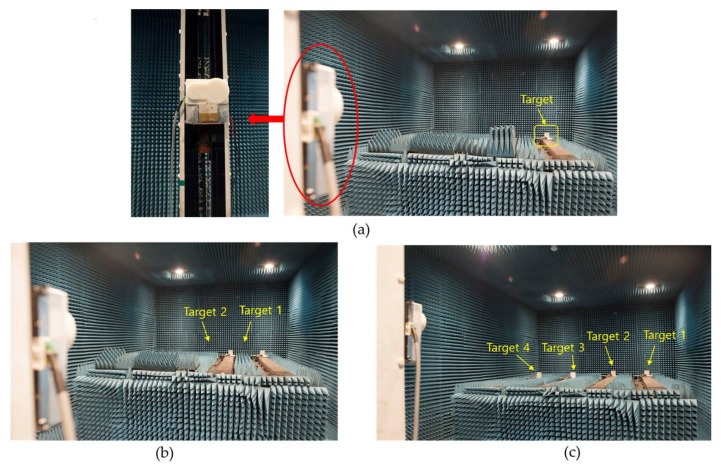
Experiment scenarios. (**a**) L-shaped radar module and scenario of one target; (**b**) Scenario of two targets; (**c**) Scenario of four targets.

A radar module with an L-shaped antenna array was set up on one side of a pillar, and the location of the radar module could be adjusted by a motor at the bottom of the pillar as shown in the left part of Figure 7a. We configured the Cartesian coordinate system following the model in Figure 1 by arranging the pillar along the *z*-axis, the L-shaped array in the *x*-*z* plane, and the inflection point of the L-shaped antenna array as the origin. Four square iron blocks with a 10-cm side length were selected as targets, and every target was mounted on one railway. The scenarios of the experiments for detecting one target, two targets, and four targets are shown in Figure 7, and the settled positions of the targets for each experiment are shown as the form (*x*, *y*, *z*) in the following list:

- 1st experiment: one target, Target (−1.2 m, 5.9 m, −0.3 m)
- 2nd experiment: two targets, Target 1 (−1.2 m, 5.9 m, −0.3 m), Target 2 (−0.4 m, 5.9 m, −0.3 m)
- 3rd experiment: two targets, Target 1 (−1.2 m, 5.9 m, −0.3 m), Target 2 (0.4 m, 4.8 m, −0.3 m)
- 4th experiment: three targets, Target 1 (−1.2 m, 5.9 m, −0.3 m), Target 2 (−0.4 m, 5.9 m, −0.3 m), Target 3 (0.4 m, 4.8 m, −0.3 m)
- 5th experiment: four targets, Target 1 (−1.2 m, 5.9 m, −0.3 m), Target 2 (−0.4 m, 5.9 m, −0.3 m), Target 3 (0.4 m, 4.8 m, −0.3 m), Target 4 (1.2 m, 4.8 m, −0.3 m)

**Figure 8:**
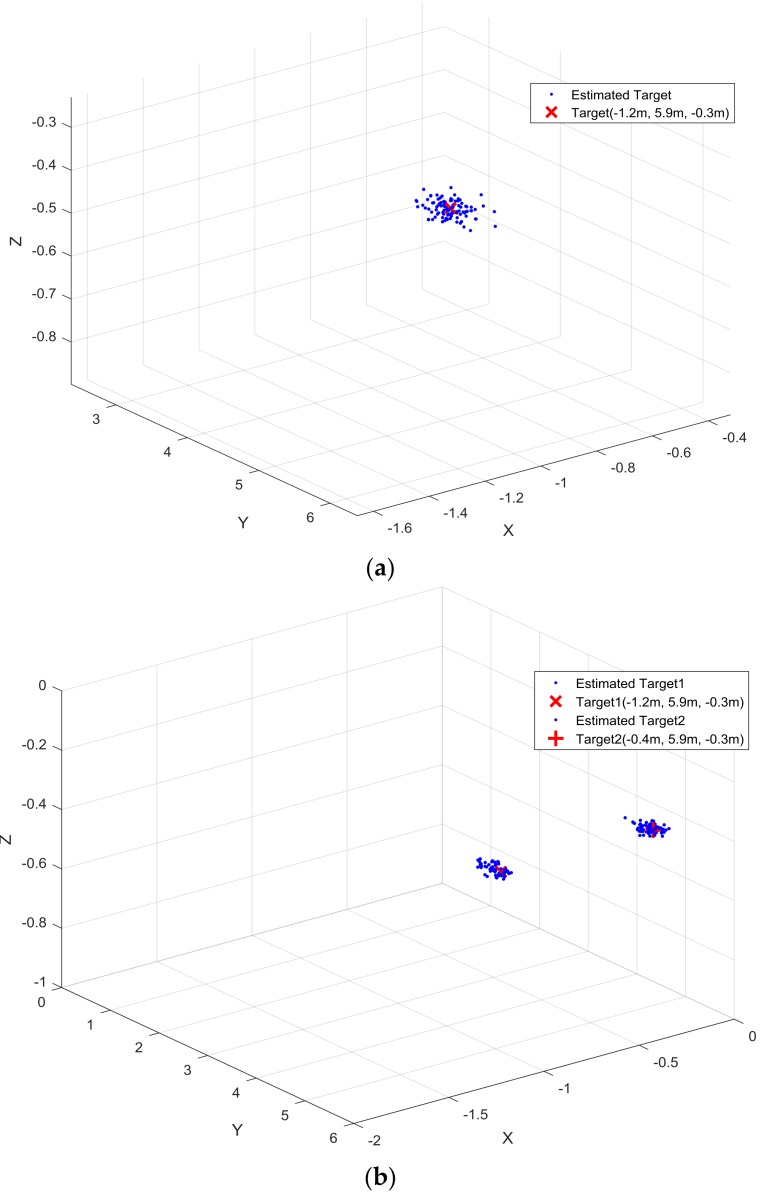

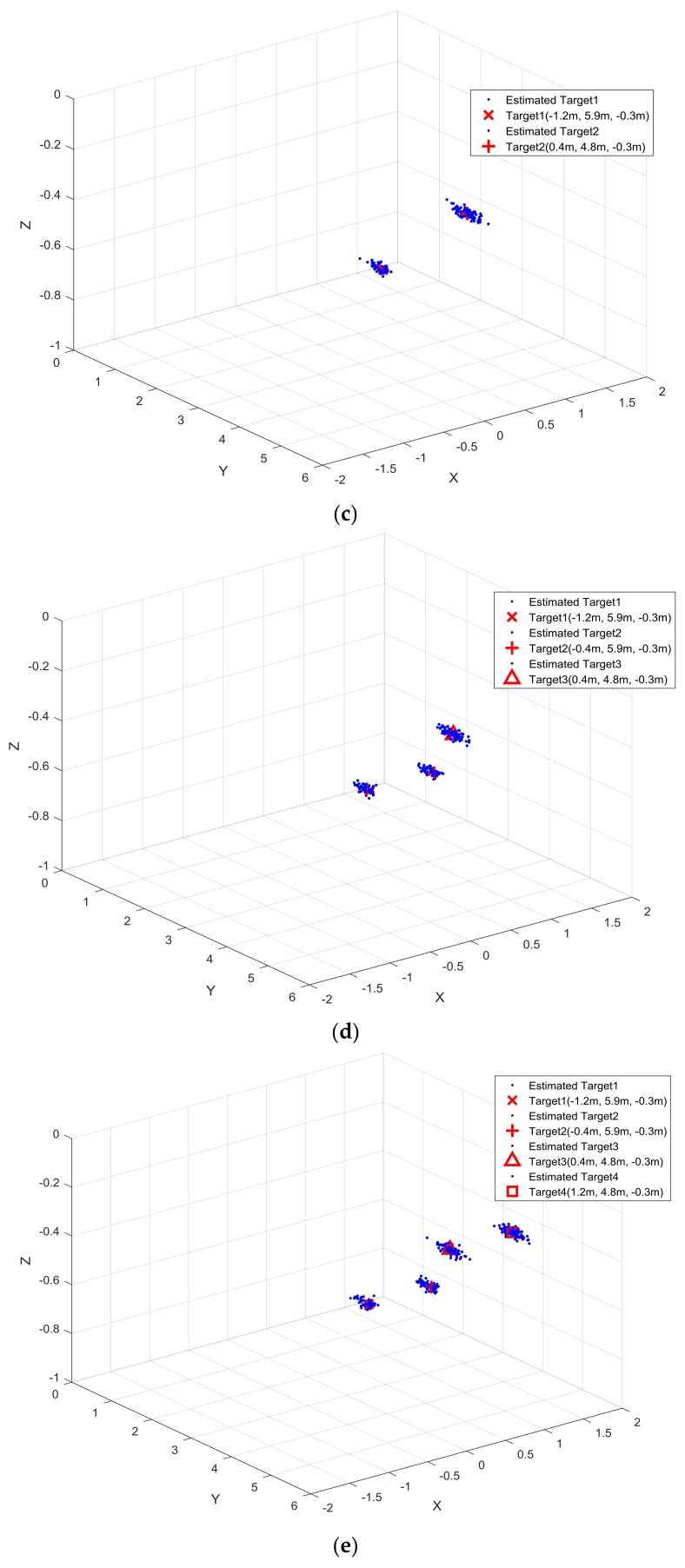
Detection results. (**a**) 1st experiment with one target; (**b**) 2nd experiment with two targets; (**c**) 3rd experiment with two targets; (**d**) 4th experiment with three targets; (**e**) 5th experiment with four targets.

We conducted 100 trials for each experiment, and the estimated positions are illustrated in Figure 8a–e, respectively. Root mean square error (RMSE) was selected as the measurement of the experiment results, and we defined the RMSE for each target as $\sqrt{\frac{1}{100}\sum_{l=1}^{100}{\left({\hat{x}}_{l}-x\right)}^{2}+{\left({\hat{y}}_{l}-y\right)}^{2}+{\left({\hat{z}}_{l}-z\right)}^{2}}$, where $\left({\hat{x}}_{l},\;{\hat{y}}_{l},\;{\hat{z}}_{l}\right)$ denotes the estimated position of the target (*x*, *y*, *z*) from the *l*-th trial. The measured RMSE values for all experiments are shown in Table 4.

In our experiments, it was firstly found that all of the five receiving channels have their own initial phases, which are different to each other due to implementation loss in the 24-GHz RF circuit. Therefore, without the estimation and compensation for the initial phases, the estimation results of the azimuth and elevation angles were biased a lot, resulting in a lot of RMSE. Thus, we tried to estimate the initial phases for each channel and use them for a calibration factor before azimuth and elevation estimation. After that, the estimation results shows less spread and less RMSE.

In the preceding section, the effect of low SNR is addressed in conjunction with subspace classification. Unlike the high SNR environment of the targets within 6 m in a chamber, the targets at a maximum of 30 m in outdoor environments are under a low SNR situation and can show performance degradation due to a low SNR and the effect of clutter.

**Figure 9:**
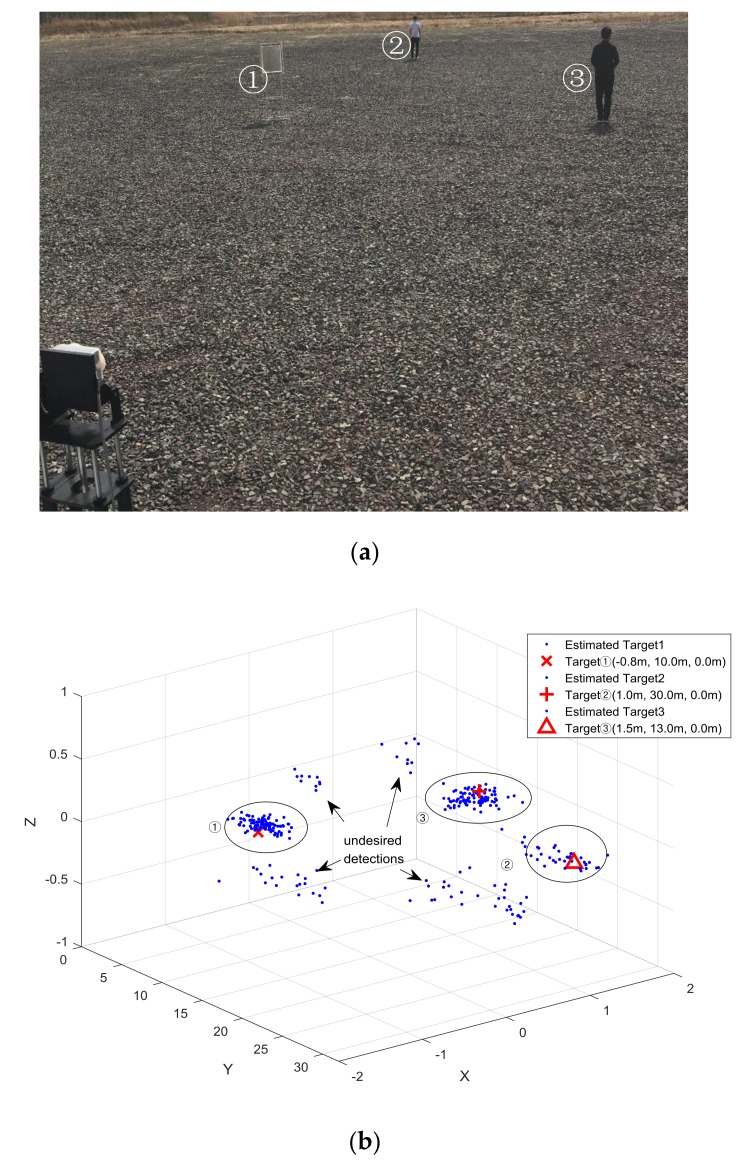
Detection results. (**a**) Outdoor experiment scenario; (**b**) detection results.

As shown in Figure 9a, the radar module was set up on a holder, and the inflection point of the L-shaped antenna array was arranged as the origin at a height of 1 m from the ground. We then arranged three targets: (1) Iron target in (−0.8 m, 10 m, 0 m); (2) Human target in (1 m, 30 m, 0 m); (3) Human target in (1.5 m, 13 m, 0 m). We tried to arrange the center of the targets at around 1 m height from the ground, and hence we put ‘0 m’ here for the three targets. It can be noted that the ground is besprinkled with little stones with various shapes, which contribute much interference for the estimations.

We conducted 100 trials for the outdoor experiment, and the estimated positions are illustrated in Figure 9b. The corresponding estimation results for each target are encompassed by the corresponding ellipse. For the first and third targets, biases in estimation results occurred due to the effect of non-Gaussian noise in the real environment. For the estimation results of the second target at a range of 30 m, the miss detection rate really increased a lot due to a low SNR. Except for the estimation results from the three targets, many undesired results were obtained from the clutter, mainly from the stones on the ground in this experiment. In the realistic scenarios, the performance of the proposed algorithms degraded due to the non-Gaussian noises and the low SNR.

## 6. Conclusions

This paper has proposed an auto-matched range-angle-Doppler 3D estimation algorithm based on a 3D shift-invariant structure and presented an FMCW radar system with L-shaped receiving antennas. Our experimental results in a chamber demonstrated that the implemented L-shaped FMCW radar system with the proposed algorithm achieved high-quality joint estimation of range, azimuth angle, and elevation angle. However, the results of the outdoor experiments demonstrated that the performance of the proposed algorithm degraded under non-Gaussian noises and the low SNR situation. Moreover, since the proposed method is composed of a variety of matrix operations such as SVD and EVD, it was difficult to implement the algorithm on FPGA and DSP for real-time estimation. Instead, the proposed algorithm is realized in MATLAB and applied with the receiving data of the experiments.

## Figures and Tables

**Table 1:** Costs of individual operations.

Operation Description	Computational Complexity
SVD of *Y*	*O*(*K*^2^*L_c_* + *K*$L_{c}^{2}\;$+${\;L}_{c}^{3}$)
EVD of *G*, *G_X_*, and *G_Y_*	*O*(*M*^3^)
$U_{s,0}^{†}$	*O*(2*M*^2^(*L_c_* – 1) + *M*^3^)
$U_{s,X\_0}^{†}$, $U_{s,Y\_0}^{†}$	*O*(2*M*^2^(*K* – 1) + *M*^3^)
Two-dimensional searching	*O*(*b*^2^*K*^2^$L_{c}^{2}$)

SVD: singular value decomposition. ***Y***: the stacked matrix in equation (13). EVD: eigenvalue decomposition. *M*: number of targets. *K*: number of antenna elements. *L_c_*: number of row of matrix in Equation (5). *b*: iteration number for two-dimensional searching. ***G***, ***G**_X_*, ***G****_Y_*, $U_{s,0}^{†}$, $U_{s,X\_0}^{†}$, $U_{s,Y\_0}^{†}$: matrices defined in Equation (35)–(37).

**Table 2:** Specifications of the developed 24-GHz transceiver and IF.

Specification	Value
Center Frequency	24.125 GHz
Frequency Bandwidth (B)	200 MHz
Frequency Period (T)	100 μs
Transmitter Output Power	20 dBm
Receiver Channel	5 ch
Receiver Noise Figure	10 dB (max)
P1dB of LNA	−15 dBm
Receiver Dynamic Range	60 dB

LNA: low noise amplifier.

**Table 3:** Specifications of the developed data-logging platform.

Submodules	Parts	Specification
FPGA	EP4SE530H35C4N	Logic element: 531,200
		Total Internal Memory: 27,376 Kbit
		Embedded Multipliers (18 × 18): 1024
DSP	TMS320C6455-1GHz	Cycle Time: 1-ns Instruction Cycle Time
		Internal Memory: 2096K-Byte
		Memory Interface: 64-Bit, Sync, Async
		DDR2 Controller: 32-Bit, 533 HMz BUS
DDR2	MT47H128M16RT	16 Meg × 16 × 8 Banks × 2EA
Flash	AT29LV040A-15TC	4 Megabit
PHY	LX971ALE	10/100 Mb/s Ethernet PHY
ADC	ADS5271	12 Bit 8 CH ADC × 2EA, 40 MHz
		LVDS Interface
RS232	MAX3221	RS-232 Line Driver/Receiver
	FT232RL	USB UART IC
CPLD	10M08SAE144C8GES	Logic element: 8000
		LABs: 500

FPGA: field programmable gate array, DSP: digital signal processor, DDR2: double data rate two, PHY: physical interface, ADC: analog to digital converter, RS232: Recommended standard 232, CPLD: complex programmable logic device, 2EA: two each, LVDS: low voltage differential signaling, USB: universal serial bus, UART: universal asynchronous receiver transmitter, IC: integrated circuit, LAB: logic array block.

**Table 4:** Measured root mean square error (RMSE) values.

Experiments	Target 1	Target 2	Target 3	Target 4
1st experiment	0.1775			
2nd experiment	0.1726	0.1649		
3rd experiment	0.1595	0.1705		
4th experiment	0.1676	0.1876	0.1658	
5th experiment	0.1692	0.1775	0.1673	0.1651
